# Properties of Milk Treated with High-Power Ultrasound and Bactofugation

**DOI:** 10.17113/ftb.59.01.21.6721

**Published:** 2021-03

**Authors:** Edita Juraga, Tomislava Vukušić Pavičić, Jasenka Gajdoš Kljusurić, Mladen Brnčić, Tomislav Juraga, Zoran Herceg

**Affiliations:** 1 ATERA d.o.o., Cehovska ulica 106, 42 000 Varaždin, Croatia; 2 Faculty of Food Technology and Biotechnology, University of Zagreb, Pierottijeva 6, 10 000 Zagreb, Croatia; 3 ATERA-ICT d.o.o., Ulica Đure Sudete 14, 42 000 Varaždin, Croatia

**Keywords:** milk properties, high-power ultrasound, bactofugation, milk treatment

## Abstract

**Research background:**

Two methods of milk treatment were used, ultrasound (innovative method) and bactofugation, after which the physicochemical and sensory properties of the milk were examined, with the primary aim of achieving the quality and consistency of the pasteurized milk.

**Experimental approach:**

Ultrasound power of 200 and 400 W and frequency of 24 kHz with constant wave cycle were used. Milk was treated for 2.5, 5, 7.5 and 10 min with sonification at 20 °C (room temperature) and thermosonification (ultrasound at temperature higher than room temperature) at 55 °C. The purpose of this study is to investigate the effect of high-power ultrasound combined with a slightly increased temperature on whole, skimmed and skimmed cow’s milk pretreated with bactofugation.

**Results and conclusions:**

The best sensory quality was achieved when milk was treated with ultrasound power of 200 W at 20 °C for max. 7.5 min. This research shows the potential of the applications of high-power ultrasound in dairy industry combined with bactofugation as a pre-treatment of milk at a slightly increased temperature (up to 55 °C).

**Novelty and scientific contribution:**

The application of these two treatments requires milder processing conditions than pasteurization, it is economical and more environmentally friendly technological process that preserves better nutritional values of milk, which is preferred by consumers.

## INTRODUCTION

Since milk is the most complete natural liquid because it contains all the substances necessary for the maintenance of health and normal functioning of the human body, it deserves special attention (*1*). According to Food and Agriculture Organization (FAO) estimates and considering the trend of further growth of the world population, gradual increase in living standards and improvement in dietary habits (*2*), it is expected that the demand and production of milk will increase at rates of 1.4% (1990-2010) to a projected growth rate of milk production of 2% by 2021. Foods available on the market are expected to have sensory properties as close as possible to the original unprocessed food in addition to the usual inevitable safety, high quality and nutritional value. Therefore, recent research has focused on the development of new food processing methods with the aim of obtaining high quality food products (*3*). Special attention is now paid to non-thermal processing methods, which include the application of high intensity ultrasound (*4*, *5*), treatment with high hydrostatic pressures, pulsed electric and magnetic fields (*6*) and non-thermal atmospheric plasma (*7*). In food treatment, high hydrostatic pressures are commercially applicable and ultrasound (US) is used in homogenization, emulsification and dispersion processes (*8*, *9*). Other applications and processes are still in the experimental phase, and in this respect further research on application of high and low ultrasound frequencies is important. The Food and Agriculture Organization (FAO) of the United Nations and the World Health Organization (WHO) define various thermal processes for the reduction and removal of microorganisms from milk (*10*, *11*). Bactofugation is used to improve the bacterial quality of raw milk. This mechanical process is mainly used in the production of cheese to eliminate anaerobic spores that can affect the flavour and destroy the texture of cheese due to uncontrolled gas formation. It is increasingly used in the production of pasteurized milk and ultra-high temperature milk (UHT), for reducing the number of heat-resistant microorganisms prior to thermal processing, all with the goal of increasing the shelf life of the milk trough a mild temperature regime. Bactofugation uses a centrifugal force to remove bacteria and spores from milk as a simple and cost-effective complement to regular pasteurization (*12*). Bactofugation of milk can remove 80-90% bacteria and 90-95% of spores (*1*). During the process, centrifugation force is gradually accelerated to achieve gentle treatment. The optimum bactofugation temperature at which the best results are achieved is 55-60 °C (*12*, *13*).

The use of high-power ultrasound has demonstrated several advantages over thermal pasteurization, such as minimizing flavour loss in juices, greater homogeneity and significant energy savings (*14*). Ultrasound improves the inactivation of microorganisms and affects enzyme activity through the effect of cavitation and is applicable to dairy products, fruits and vegetables (*15*, *16*). The advantages of ultrasound over sterilization are minimal loss of flavour, better homogenization and significant energy savings (*17*). A combination of ultrasound with high pressure, heat or pH change has been shown to be an effective method of killing microorganisms due to the effect of the produced free radicals, generated heat and resulting shear forces (*18*). However, it is necessary to pay attention to the proper application of ultrasound, as too high ultrasonic power can cause drastic changes in milk fat composition, resulting in a bitter, tasteless liquid due to the oxidation of fat (*18*). Proper application of ultrasound requires the use of appropriate power, amplitude of the sound wave and ultrasound frequency, as well as the optimum treatment time at lower temperatures to avoid undesirable changes in the treated material. Therefore, the aim of this work is to investigate the possibility of processing raw whole milk, skimmed milk and previously bactofuged skimmed milk by applying high-power ultrasound in combination with a slightly elevated temperature in order to achieve the same microbiological acceptability as with pasteurization and to monitor the possible effects of the two treatments (ultrasound and bactofugation) on the chemical composition and sensory properties of the milk.

## MATERIALS AND METHODS

**Fig. S1:**
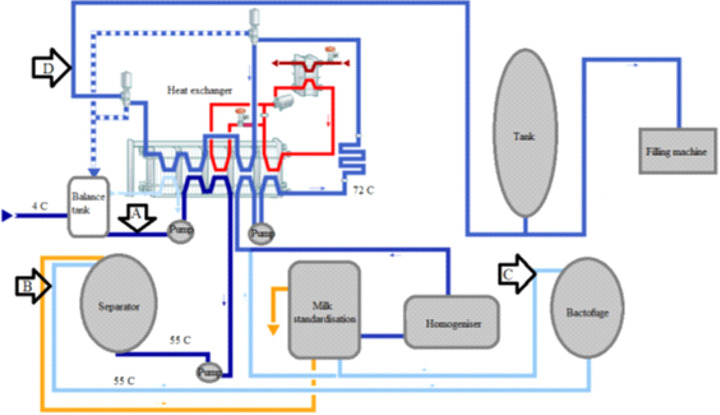
Sampling locations of the same treatment batch: A=before the separator (raw whole milk), B=after the separator (skimmed milk), C=after the bactofuge (skimmed bactofuged milk), and D=at the outlet from the heat exchanger (pasteurized milk as a reference sample)

Milk samples were collected aseptically from a Croatian dairy industry in sterile vials from the sampling valves before the separator (raw whole milk, A), after the separator (skimmed milk, B), after the bactofuge (skimmed bactofuged milk, C), and the final sample was the milk pasteurized by the classical high-temperature short-time method (72 °C/15 s) used as reference sample (D) for each experiment, as shown in Fig. S1. Samples from each batch were analyzed in triplicate.

### Processing of milk with ultrasonic processor

Ultrasonic processor used in this study was model UP 400S, Hielscher Ultrasoniscs GmbH, Teltow, Germany, with: effective output power 400 W, voltage 230 V, 10-100% ultrasound cycle, ultrasonic frequency 24 kHz and amplitude 12-260 μm. We used a 7-mm diameter titanium probe immersed at a depth of 2 cm in each sample of milk.

Four experimental treatments were designed with different applied ultrasound power (at the same frequency 24 kHz) and temperature during the transduction, as follows: experiment A: *P*=200 W, *t*=20 °C, experiment B: *P*=200 W, *t*=55 °C, experiment C: *P*=400 W, *t*=20 °C, and experiment D: *P*=400 W, *t*=55 °C.

### Design of ultrasound treatment

Within each of the four experimental treatments, ultrasonic processing was performed on three different samples of milk: on the raw whole milk, skimmed milk and skimmed bactofuged milk, while pasteurized samples were used as a reference (*19*). Each of the three different milk samples was treated during four time periods (2.5, 5, 7.5 and 10 min). Thus, 12 treatments (3 milk samples×4 time periods) were performed in each experiment (A-D), including four control samples (raw whole milk, skimmed milk, skimmed bactofuged milk and pasteurized milk), hence a total of we analysed 64 samples (4×12 treated and 4×4 reference samples). All trials were performed as three independent measurements, and the results represent the mean value of all three measurements. In order to carry out the statistical analysis, the applied multivariate tools showed the influence of the main components and the correlation of the values ​​of all the experiments in which the treatment (T) conditions are observed (process parameters: *P=*0, 200 or 400 W, *ν*=0 or 24 kHz, *t*=20 or 55 °C and time=0, 2.5, 5, 7.5 or 10 min), treatments T1-T4 (*P*=200 or 400 W, *ν*=24 kHz, *t*=20 or 55 °C) and time=2.5, 5, 7.5 or 10 min) are raw whole milk treated for 2.5 (T1), 5 (T2), 7.5 (T3) and 10 (T4) min, treatments T5-T8 (*P*=200 or 400 W, *ν*=24 kHz, *t*=20 or 55 °C and time=2.5, 5, 7.5 or 10 min) are skimmed milk treated for 2.5 (T5), 5 (T6), 7.5 (T7) and 10 (T8) min, and treatments T9-T12 (*P*=200 or 400 W, *ν=*0 or 24 kHz, *t*=20 or 55 °C and time=0, 2.5, 5, 7.5 or 10 min) are skimmed bactofuged milk treated for 2.5 (T9), 5 (T10), 7.5 (T11) and 10 (T12) min. The process parameters *P, ν, t* and time varied in each conducted experiment (experiment A: *P*=200 W, *t*=20 °C, experiment B: *P*=200 W, *t*=55 °C, experiment C: *P*=400 W, *t*=20 °C, and experiment D: *P*=400 W, *t*=55 °C).

### The acidity of milk

Physical quality of all milk samples before and immediately after the treatment was measured. Titratable acidity and active acidity of the samples was monitored. We used the standard Soxhlet-Henkel method for determining the titration acidity of milk (*1*). Reagents used for the analysis were 0.1 M NaOH solution, 2% alcoholic solution of phenolphthalein, and 5% cobalt sulfate solution (CoSO_4_·7H_2_O). Cobalt sulfate mixed with 50 mL milk was the reference pink. Briefly, 1 mL of phenolphthalein indicator was added to 20 mL tempered milk sample at 20 °C, the mixture was stirred and quickly titrated with 0.1 M NaOH with continuous stirring until the colour changed to a light pink that was stable for one min and compared to the prepared standard pink colour (a mixture of 1 mL of 5% cobalt sulfate solution and 20 mL milk freshly prepared, no longer than 3 h before titration). The milk sample was titrated with 0.1 M NaOH and milk acidity was calculated according to the formula:


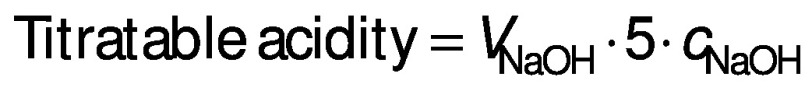


where *V*_NaOH_ is the volume of 0.1 M NaOH (in mL) used to neutralize 20 mL of the test sample, 5 is a constant, and *c*_NaOH_=0.1 mol/L. The results are expressed in Soxhlet-Henkel degrees (°SH) as the arithmetic mean of three parallel analyses. The maximum permissible difference in triple milk analyses was 0.2 °SH. If larger difference is encountered, the analysis was repeated.

The pH was measured using a pH meter model 225 (Mettler Toledo, Munich, Germany) with an integrated temperature compensation. The electrodes were immersed in a sample of milk that was lightly stirred until the value of the display remained stable. For the same sample, three measurements were performed with washing and wiping of the electrode between measurements, and the result was the arithmetic mean.

### Determination of the chemical composition of milk samples

The composition of all milk samples before and immediately after the treatment was analyzed. The mass fractions (*w*/(g/100 g) of total solids, non-fat solids, milk fat, protein and lactose were determined using the IR spectroscopy (*20*) and MilkoScan 4000 (Type 71200; Foss Electric A/S, Copenhagen, Denmark) instrument, while calcium mass fraction was determined with the titrimetric method (*21*). A volume of 2.5 mL milk was supplemented with 100 mL distilled water. The solution was transferred to an Erlenmeyer flask in which 2 mL of 10 M NaOH were added and the mixture was stirred on a magnetic stirrer for 2-3 min. Two drops of indicator calcon-carbonic acid were added and the mixture was titrated with a 0.05 M EDTA solution with stirring until the light red colour changed to light blue.

To determine the accuracy of the titration, 0.25 mL of 0.05 M CaCl_2_ was added to the titrated solution and the solution turned red again, then it was titrated once again with 0.05 M EDTA solution until the colour changed to light blue. The calcium mass (in mg) in 100 g milk was calculated according to the following formula:


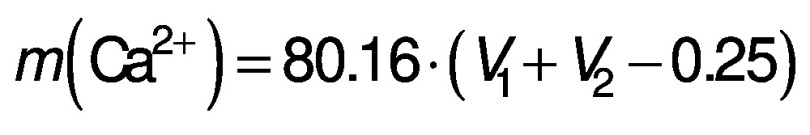


where 80.16 and 0.25 are constants, *V*_1_ is the volume of EDTA solution used for the first titration (mL), *V*_2_ is the volume of EDTA solution used for second titration (mL).

### Sensory evaluation of milk samples

Sensory examination included an assessment of the appearance (max. 3 points), colour (max. 2 points), odour (max. 3 points) and taste (max. 12 points) of milk. The analysis of appearance included texture, consistency and possible deposition, and the taste included standard flavours and aromas based on the IDF Standards (*22*).

The sensory evaluation was conducted by a committee consisting of five certified sensory analysts for milk and dairy products (mean age 35.7, two men and three women). All samples were analyzed at 20 °C in labelled glass cups. The odour was evaluated first. When evaluating the colour of the samples, the reference samples of the original colour of whole and skimmed milk were used, in fully transparent glasses. The precipitate and the consistency of milk were controlled by pouring the milk into another glass cup down the wall to make it easy to observe possible flakes and bruises created by the grain.

For the evaluation of the analysis, the scoring method was used with a maximum of 20 points (*22*). In the cases when the grade of individual sensory properties in the examined sample decreased by ±1 point, the mean was taken, and when it deviated by more than 1 point the analysis was repeated. The disqualified sample on the sensor was the one that received 0 points for any property.

### Statistical data processing

Descriptive statistics was used to obtain mean values, standard errors and minimum and maximum values ​​for each treatment and separately for each experiment (*23*). In the examination of similarities or differences in the data for each observed characteristic (chemical, physical parameter and sensory evaluation), *t-*test methods were used for different treatments and experiments with the chosen level of significance (risk) 0.05 (95%). For the purposes of linking, *i.e.* establishing similarities and/or differences in a large set of data for each observed feature (treatment by experiment), multivariate statistical methods were applied on the same set of data as well as on the data not belonging to the same set (*24*).

Factor analysis is the generic name given to the class of multivariate statistical methods whose primary purpose is to define the basic structure in the data matrix. In general, it deals with the problem of analyzing the correlation structure between a large number of variables (*e.g.* sensory test, physicochemical measurements, *etc.*) by defining a set of common dimensions known as factors (*24*). In factor analysis, factors are formed by maximizing their explanation of a whole set of variables.

We applied the principal component analysis (PCA) to confirm the grouping, and the analysis of the main components also provided a pictorial representation to determine the reasons why groups were formed in a particular manner. Multivariate analysis (chemometrics) proved to be a powerful tool for identifying the data in the experimental part (*25*, *26*).

Among the chemometric methods, the principal component analysis served to identify the experimental data and group them based on their similarity and variety. First, the data were organized in the matrix with the treatments set in rows and experiments located in the columns (*27*). Each variable vector was automatically scaled with the corresponding wavelength of the sample and displayed as:


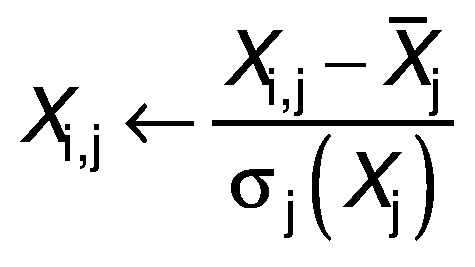


The scaled matrix of data *X* is approximated with projections in the subsystem of the main components P:


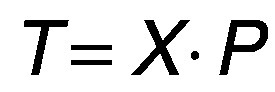


Data processing software STATISTICA v. 8 (*28*) was used for data processing.

## RESULTS AND DISCUSSION

The research was carried out on milk sampled from the production line at various processing stages. Raw whole milk, skimmed milk and skimmed milk after bactofugation were used. The effect of pretreatment of milk by bactofugation has been investigated, as we assumed that microbiological results of sonification of previously bactofuged milk would be more significant and that this combination might extend the shelf life of milk. The centrifugal separator of bacteria (bactofuge) can isolate about 80-90% of the bacteria and 90-95% of the spores from the milk, so it is to be expected that such a reduced number of remaining bacteria in the bactofuged milk would be successfully inactivated by ultrasound. The nominal power used on 200 mL of milk was 200 and 400 W at 24 kHz. The treated samples were measured after 2.5, 5, 7.5 or 10 min, and untreated reference samples were used for comparison between the same batch and milk group (raw whole milk, skimmed milk and skimmed bactofuged milk).

The milk treated with ultrasound for too long had an unpleasant taste, which can be explained by the study of Zabbia *et al.* (*29*) where the effect of treatment of milk with high intensity ultrasound resulted in the formation of certain volatile components causing a change of taste and the appearance of unpleasant odour. Furthermore, it has been demonstrated that ultrasonic processing of milk at temperatures higher than 60 °C resulted in denaturation of whey proteins (*30*, *31*).

With a large number of experimental data collected (physical quality, chemical composition and analysis of sensory properties of treated and untreated milk samples), it is very difficult to determine the changes in different milk samples under different conditions of treatment (power, frequency, temperature and time). In such complex systems, multivariate analysis of variance (MANOVA) is used to determine differences in the observed set of data (*26*).

Table 1 shows the results of the MANOVA analysis how treatment variations affect the titratable acidity and pH values. The multivariate analysis showed that the power of 400 W influenced the change in mean values ​​of titratable acidity (6.78 °SH) and pH (6.680) at a significance level of α=0.05. The frequency change did not affect the value of titratable acidity, but the frequency of 24 kHz used with a power of 400 W affected the pH change (6.692). Multivariate analysis suggests that the temperature change is significant for the samples heated to 55 and 72 °C for titratable acidity and pH. The processing of milk by high-power ultrasound does not lead to significant pH changes (*32*). Shanmugam *et al.* (*33*) came to the same conclusions using 12-mm diameter ultrasonic probes, 450 W and 20 kHz frequency, skimmed milk with 1.5% fat and time of 15, 30, 45 and 60 min. Walstra *et al.* (*34*) found that ultrasonic processing may lower the pH value of milk due to the hydrolysis of phosphoric esters because ultrasound mediates in enzymatic reactions of some enzymes and cavitation can accelerate some chemical reactions causing a fall in the pH. Therefore, this may be the cause of the pH drop at 400 W, 24 kHz and 20 °C (Table 1). The average protein mass fraction in milk is 3.4 g/100 g and ranges from 2.9 to 5.0 (*14*). Under different experimental conditions (200 or 400 W, frequency of 24 kHz, temperature 20 and 55 °C) and different treatment times (2.5, 5, 7.5 and 10 min), the influence of ultrasound on the change of the total solids, non-fat solids, fat and protein mass fractions was not observed, which is consistent with the conclusions reached by Cameron *et al.* (*35*). After the ultrasound treatment of milk, Chemat *et al.* (*36*) noted an increase in fat content as a result of the breakdown of fat-aggregate membranes under the influence of ultrasound.

**Table 1:** Multivariate analysis (MANOVA) of the effect of experimental treatments (ultrasound power, frequency, temperature and time) on the changes in physical properties of milk samples. Results are compared with untreated samples

Condition			Titratable acidity/°SH		pH
*P*/W	0	(6.50±0.08)^a^		(6.71±0.01)^a^	
	200	(6.63±0.07)^a^		(6.70±0.01)^a^	
	400	(6.78±0.07)^b^		(6.68±0.01)^b^	
*ν*/kHz	0	(6.5±0.08)^a^		(6.71±0.01)^a^	
	24	(6.70±0.05)^a^		(6.69±0.01)^b^	
*t*/°C	20	(6.81±0.05)^a^		(6.71±0.01)^a^	
	55	(6.58±0.06)^b^		(6.69±0.01)^b^	
	72	(6.40±0.14)^b^		(6.72±0.02)^b^	
time/min	0	(6.61±0.08)^a^		(6.71±0.01)^a^	
	2.5	(6.59±0.08)^a^		(6.71±0.01)^a^	
	5	(6.67±0.08)^a^		(6.70±0.01)^a^	
	7.5	(6.744±0.08)^a^		(6.70±0.01)^a^	
	10	(6.800±0.08)^a^		(6.69±0.01)^a^	

Data are presented as mean value±standard error (SE), *N*=3. Values ​​with different letters in superscript in the same column differ at a significance level of 5%

The aforementioned effect, however, exceptionally low increase in fat content, can be observed in the present study when the whole milk is processed by ultrasound at 200 W (experiment A) and 400 W (experiments C and D). Average values of the milk composition and physical characteristics are presented in Table 2.

**Table 2:** Average values of composition and physical characteristics of differently treated milk

	Treatment	*t*/20 °C											*t*/55 °C											
			*w*/(g/100 g)					*w*/(mg/100 g)							*w*/(g/100 g)							*w*/(mg/100 g)		
	Titratable acidity/°SH	pH	TS	SNF	Fat	Protein	Lactose	Ca			Titratable acidity/°SH		pH	TS		SNF		Fat	Protein		Lactose		Ca	
*P=*200 W	SM	6.4	6.7	13.0	8.7	4.3	3.4	4.6	121.8			6.4		6.7	12.5			8.2	4.3	3.2	4.3		113.8	
	T1	6.2*	6.7	12.9	8.6	4.3	3.4	4.5	122.2			6.4		6.7	12.2			8.2	4.0	3.2	4.3		116.2	
	T2	6.2*	6.8	12.9	8.7	4.3	3.4	4.6	122.2			6.8		6.7	12.2			8.2	4.0	3.2	4.3		116.2	
	T3	6.2*	6.7	13.0	8.7	4.3	3.4	4.6	122.9			6.6		6.7	12.2			8.2	4.0	3.2	4.3		118.3	
	T4	7.0	6.7	13.0	8.7	4.3	3.5	4.6	122.9			6.6		6.6	12.3			8.2	4.0	3.2	4.3		120.2	
	RM	6.8	6.8	9.7*	9.4	0.4*	3.9	4.8	132.3*			6.6		6.7	9.1^#^			8.8	0.1^#^	3.6	4.5		128.9	
	T5	7	6.8	9.7*	9.3	0.4*	3.9	4.8	132.2*			6.8		6.7	9.1^#^			8.8	0.1^#^	3.6	4.5		132.3^#^	
	T6	6.6	6.8	9.7*	9.3	0.5*	3.8	4.8	133.1*			6.8		6.7	9.1^#^			8.8	0.1^#^	3.6	4.5		130.2	
	T7	6.8	6.8	9.7*	9.4	0.4*	3.9	4.8	133.4*			6.6		6.7	8.8^#^			8.7	0.1^#^	3.6	4.5		133.2^#^	
	T8	6.8	6.8	9.7*	9.3	0.5*	3.8	4.8	133.4*			6.6		6.7	9.1^#^			8.8	0.1^#^	3.6	4.5		133.9^#^	
	BF	6.8	6.8	9.4*	9.0	0.5*	3.6	4.8	122.6			6.6		6.6	8.9^#^			8.6	0.1^#^	3.4	4.5		117.0	
	T9	6.8	6.8	9.4*	9.0	0.5*	3.6	4.8	123.0			6.4		6.7	8.8^#^			8.6	0.1^#^	3.4	4.5		120.2	
	T10	6.8	6.8	9.5*	9.0	0.5*	3.6	4.8	122.9			6.2^#^		6.7	8.8^#^			8.6	0.1^#^	3.4	4.5		118.6	
	T11	6.8	6.8	9.4*	9.0	0.5*	3.6	4.8	123.2			6.6		6.7	8.9^#^			8.6	0.1^#^	3.4	4.5		120.7	
	T12	6.8	6.8	9.5*	9.0	0.5*	3.6	4.8	124.1			6.6		6.7	8.9^#^			8.6	0.1^#^	3.3	4.5		121.8	
	P	6.6	6.8	10.5*	9.6	0.9*	3.7	5.0	121.0			6.2^#^		6.7	10.2			9.2	0.9^#^	3.3	4.9		114.6	
*P=*400 W	SM	6.8	6.7	12.8	8.7	4.2	3.4	4.6	119.4			6.4		6.7	12.4			8.4	3.9	3.3	4.5		123.4	
	T1	6.6	6.6	12.9	8.7	4.2	3.4	4.6	120.2			6.4		6.7	12.4			8.5	3.9	3.3	4.5		122.3	
	T2	7.8*	6.5	13.0	8.8	4.2	3.5	4.6	119.2			6.2		6.7	12.5			8.4	4.1	3.3	4.5		122.4	
	T3	8.4*	6.5	13.0	8.9	4.3	3.6	4.6	119.4			6.0		6.7	12.4			8.4	4.0	3.3	4.5		120.2	
	T4	8.6*	6.5	13.2	8.9	4.4	3.6	4.6	120.2			6.0		6.7	12.3			8.3	4.0	3.2	4.4		117.8	
	RM	6.6	6.7	12.8	8.7	0.5*	3.8	4.8	121.0			6.5		6.7	9.3			9.0	0.1^#^	3.6	4.7		124.3	
	T5	6.6	6.7	12.9	8.7	0.5*	3.7	4.7	122.4			6.2		6.7	9.3			9.0	0.1^#^	3.6	4.7		120.2	
	T6	7	6.7	13.0	8.8	0.5*	3.8	4.8	121.2			6.6		6.7	9.3			9.0	0.1^#^	3.6	4.7		122.8	
	T7	7.2	6.7	13.0	8.9	0.4*	3.8	4.8	122.6			6.8		6.7	9.3			9.0	0.1^#^	3.6	4.7		123.0	
	T8	7.4	6.7	13.2	8.9	0.4*	3.8	4.8	121.8			6.4		6.7	9.3			9.0	0.1^#^	3.6	4.7		124.2	
	BF	6.8	6.7	9.5*	9.0	0.5*	3.5	4.8	123.4			6.4		6.7	9.1^#^			8.8	0.1^#^	3.4	4.7		123.3	
	T9	6.8	6.7	9.5*	9.0	0.5*	3.6	4.8	125.0			6.2		6.7	9.1^#^			8.7	0.1^#^	3.4	4.7		122.6	
	T10	6.8	6.7	9.5*	9.0	0.5*	3.6	4.8	123.4			6.2		6.7	9.0^#^			8.7	0.0^#^	3.4	4.6		123.0	
	T11	6.8	6.7	9.5*	9.1	0.4*	3.5	4.8	120.2			6.2		6.7	9.1^#^			8.7	0.1^#^	3.4	4.7		123.1	
	T12	6.6	6.7	9.4*	9.0	0.5*	3.5	4.8	122.6			6.8		6.7	9.0^#^			8.7	0.0^#^	3.4	4.6		122.6	
	P	6.8	6.7	9.7*	9.2	0.5*	3.7	5.0	122.6			6.0		6.7	10.3			9.2	1.1^#^	3.4	4.9		129.1	

* and ^#^ for the same experiment (C or D): significantly different characteristics (significance level of 5%) of differently treated milk (RM=raw whole milk, SM=skimmed milk, BF=skimmed bactofuged milk and P=pasteurized milk), TS=Total solids, SNF=non-fat solids, T1-T4=raw whole milk treated for 2.5 (T1), 5 (T2), 7.5 (T3) and 10 (T4) min, T5-T8= skimmed milk treated for 2.5 (T5), 5 (T6), 7.5 (T7) and 10 (T8) min and T9-T12=skimmed bactofuged milk treated for 2.5 (T9), 5 (T10), 7.5 (T11) and 10 (T12) min

The multivariate analysis of the chemical composition of milk samples (Table 3) treated with different intensities of ultrasound showed very low standard error (SE) for lactose. The difference in the lactose content was significant at 5% significance level in pasteurized samples (a temperature of 72 °C and a time of 15 s) at a power of 200 W and a frequency of 24 kHz.

**Table 3:** Results of multivariate analysis (MANOVA) of the effect of experimental treatments (ultrasound power, frequency, temperature and time) on the changes in chemical composition of milk samples. Results are compared with untreated samples (condition: *P*, *ν, t and* time =0)

Condition			TS		SNF		Fat		Protein		Lactose		Ca	
*P*/W	0	(10.5±0.4)^a^		(9.0±0.1)^a^		(1.2±0.5)^a^		(3.49±0.05)^a^		(4.8±0.0)^a^		(123.7±1.4)^a^	
	200	(10.4±0.4)^a^		(8.8±0.1)^a^		(1.6±0.4)^a^		(3.49±0.04)^a^		(4.6±0.0)^b^		(124.9±1.2)^a^	
	400	(11.0±0.4)^a^		(8.8±0.1)^a^		(1.5±0.4)^a^		(3.51±0.04)^a^		(4.7±0.0)^a^		(121.8±1.2)^a^	
*ν*/kHz	0	(10.5±0.4)^a^		(9.0±0.1)^a^		(1.2±0.5)^a^		(3.49±0.05)^a^		(4.8±0.0)^a^		(123.7±1.4)^a^	
	24	(10.7±0.3)^a^		(8.8±0.0)^a^		(1.6±0.3)^a^		(3.50±0.03)^a^		(4.6±0.0)^b^		(123.3±0.9)^a^	
*t*/°C	20	(10.9±0.28)^a^		(8.9±0.0)^a^		(1.7±0.3)^a^		(3.54±0.03)^a^		(4.7±0.0)^a^		(127.3±0.9)^a^	
	55	(10.2±0.3)^a^		(8.7±0.1)^a^		(1.4±0.3)^a^		(3.44±0.03)^a^		(4.6±0.0)^a^		(123.5±1.0)^a^	
	72	(10.4±0.7)^a^		(9.2±0.1)^b^		(0.9±0.8)^a^		(3.49±0.08)^a^		(4.9±0.0)^b^		(122.9±2.5)^a^	
time/min	0	(10.6±0.4)^a^		(8.8±0.1)^a^		(1.6±0.5)^a^		(3.49±0.05)^a^		(4.6±0.0)^a^		(124.6±1.4)^a^	
	2.5	(10.6±0.4)^a^		(8.8±0.1)^a^		(1.5±0.5)^a^		(3.48±0.05)^a^		(4.6±0.0)^a^		(125.1±1.4)^a^	
	5	(10.6±0.4)^a^		(8.8±0.1)^a^		(1.5±0.5)^a^		(3.49±0.05)^a^		(4.6±0.0)^a^		(125.1±1.4)^a^	
	7.5	(10.6±0.4)^a^		(8.8±0.1)^a^		(1.5±0.5)^a^		(3.50±0.05)^a^		(4.6±0.0)^a^		(125.8±1.4)^a^	
	10	(10.6 0.4)^a^		(8.8±0.1)^a^		(1.4±0.5)^a^		(3.49±0.05)^a^		(4.6±0.0)^a^		(126.2±1.4)^a^	

Data are presented as mean value±standard error (SE), *N*=3. Values ​​with different letters in superscript in the same column differ at a significance level of 5%. TS=total solids, SNF=non-fat solids, Ca=calcium

A study of the effect of ultrasound on the chemical composition of milk concluded that the ultrasound does not have a significant influence on the lactose content (*35*). However, using a multivariate analysis, lower lactose content was determined under experimental conditions of 200 W, 24 kHz and 55 °C (experiment B), as a result of the quality of the milk used for a particular batch. The composition of milk is variable, the average lactose mass fraction in milk is 4.8 g/100 g, ranging from 3.6 to 5.5 (*14*, *37*). Samples treated at 200 W, 24 kHz and 55 °C showed a mild change of odour in the sense that freshness of whole milk was affected by 7.5 min of processing and of skimmed bactofuged milk by 2.5 and 10 min of processing. These parameters (200 W, 24 kHz, 55 °C) influenced the taste of all whole milk samples in which a change in the characteristic taste of milk with mild taste was burnt, while in the sample of bactofuged skimmed milk treated for 10 min the loss of freshness was observable. Treatment of whole milk at 400 W and 24 kHz at room temperature for 5 and 10 min caused a change of odour due to the loss of freshness. Zabbia *et al.* (*29*) concluded that prolonged treatment leads to the appearance of a large number of volatile components and results in the change of taste. The results in this paper (Table 4 and Table 5) are consistent with those findings.

**Table 4:** Average values of sensorial properties for experimental treatments A, B, C and D for differently treated milk samples

Sensorial property	Experiment	Treatment															
SM	T1	T2	T3	T4	RM	T5	T6	T7	T8	BF	T9	T10	T11	T12	P
Odour (*N*_max_=3)	A	3^a^	3^a^	3^a^	3^a^	2.5^a^	3^a^	3^a^	3^a^	3^a^	3^a^	3^a^	3^a^	3^a^	3^a^	1.5^b^	3^a^
	B	3^a^	3^a^	3^a^	2.5^a^	3^a^	3^a^	3^a^	3^a^	3^a^	3^a^	3^a^	2.5^a^	3^a^	3^a^	2.5^a^	3^a^
	C	3^a^	3^a^	1^b^	0^b^	1^b^	3^a^	3^a^	3^a^	3^a^	1.5^b^	3^a^	3^a^	3^a^	3^a^	3^a^	3^a^
	D	3^a^	2.5^a^	3^a^	2.5^a^	2^a^	3^a^	3^a^	3^a^	3^a^	2.5^a^	3^a^	3^a^	3^a^	3^a^	3^a^	3^a^
Taste (*N*_max_=12)	A	12^a^	11.5^a^	12^a^	9.5^a^	6^a^	11^a^	11^a^	7.5^a^	8^a^	7.5^a,b^	11^a^	11^a^	7^a^	9^a^	5.5^a^	11^a^
	B	11^a^	6^b^	6^b^	6.5^b^	6.5^a^	11.5^a^	11.5^a^	11^b^	10^b^	11^b^	11^a^	9.5^b^	11^b^	9^a^	8.5^a^	11.5^a^
	C	12^a^	12^a^	3^c^	2.5^c^	3^b^	12^a^	9^a^	7^a^	5.5^a^	5^b^	12^a^	12^a^	9^a^	7.5^a^	7^a^	12^a^
	D	12^a^	9^c^	9.5^a^	7.5^a,b^	6^a^	11^a^	11^a^	9.5^b^	10^b^	6^b^	11.5^a^	11^a^	11.5^b^	9.5^a^	7^a^	10^a^
Total (*N*_max_=20)	A	20^a^	19.5^a^	20^a^	17.5^a^	13.5^a^	19^a^	19^a^	15.5^a^	16^ab^	15.5^a^	19^a^	19^a^	15^a^	17^a^	12^a^	19^a^
	B	19^a^	14^b^	14^b^	14^a^	14^a^	19.5^a^	19.5^a^	19^a^	18^a^	19^a^	19^a^	17^a^	19^b^	17^a^	16^b^	19.5^a^
	C	20^a^	20^a^	9^c^	7.5^b^	8^b^	20^a^	17^a^	15^a^	13.5^b^	11.5^b^	20^a^	20^a^	17^b^	15.5^a^	15^b^	20^a^
	D	20^a^	16.5^ab^	17.5^a^	15^a^	12.5^a^	19^a^	19^a^	17.5^a^	18^a^	13.5^b^	19.5^a^	19^a^	19.5^b^	17.5^a^	15^b^	18^a^

Values in the same column marked with different letters in superscript differ at a significance level of 5%. RM=raw whole milk, SM=skimmed milk, BF=skimmed bactofuged milk and P=pasteurized milk, T1-T4=raw whole milk treated for 2.5 (T1), 5 (T2), 7.5 (T3) and 10 (T4) min, T5-T8= skimmed milk treated for 2.5 (T5), 5 (T6), 7.5 (T7) and 10 (T8) min and T9-T12=skimmed bactofuged milk treated for 2.5 (T9), 5 (T10), 7.5 (T11) and 10 (T12) min. Experimental treatment: A (*P*=200 W, *t*=20 °C), B (*P*=200 W, *t*=55 °C), C (*P*=400 W, *t*=20 °C) and D (*P*=400 W, *t*=55 °C)

**Table 5:** Results of multivariate analysis (MANOVA) on the sensory properties based on the effect of experimental treatments (ultrasound power, frequency, temperature and time) on changes in sensory properties of milk samples. Results are compared with untreated samples (condition: *P*, *ν, t and* time =0)

Condition		Appearance	Colour	Odour	Taste	Total
*P*/W	0	(3.00±0.03)^a^	(2.00±0.00)^a^	(3.0±0.1)^a^	(11.3±0.4)^a^	(19.3±0.5)^a^
	200	(2.98±0.02)^a^	(2.00±0.00)^a^	(2.9±0.1)^a^	(8.8±0.3)^b^	(16.7±0.4)^b^
	400	(2.94±0.02)^a^	(2.00±0.00)^a^	(2.5±0.1)^b^	(7.9±0.3)^c^	(15.4±0.4)^b^
*ν*/kHz	0	(3.00±0.03)^a^	(2.00±0.00)^a^	(3.0±0.1)^a^	(11.3±0.4)^a^	(19.3±0.5)^a^
	24	(2.96±0.02)^a^	(2.00±0.00)^a^	(2.7±0.1)^b^	(8.4±0.2)^c^	(16.0±0.3)^b^
*t*/°C	20	(2.97±0.02)^a^	(2.00±0.00)^a^	(2.7±0.1)^a^	(8.5±0.2)^a^	(16.1±0.3)^a^
	55	(2.97±0.02)^a^	(2.00±0.00)^a^	(2.5±0.1)^a^	(9.2±0.3)^a^	(16.8±0.3)^a^
	72	(3.00±0.05)^a^	(2.00±0.00)^a^	(3.0±0.2)^b^	(11.2±0.7)^b^	(19.2±0.8)^b^
time/min	0	(3.00±0.03)^a^	(2.00±0.00)^a^	(3.0±0.1)^a^	(11.4±0.4)^a^	19.4±0.5)^a^
	2.5	(3.00±0.03)^a^	(2.00±0.00)^a^	(2.9±0.1)^a^	(10.3±0.4)^a^	(18.1±0.5)^a^
	5	(3.00±0.03)^a^	(2.00±0.00)^a^	(2.8±0.1)^a^	(9.1±0.4)^b^	(16.9±0.5)^b^
	7.5	(3.00±0.03)^a^	(2.00±0.00)^a^	(2.6±0.1)^b^	(8.3±0.4)^c^	(15.8±0.5)^c^
	10	(2.89±0.03)^a^	(2.00±0.00)^a^	(2.3±0.1)^b^	(7.3±0.4)^d^	(14.4±0.5)^d^

Data are presented as mean value±SE, SE=standard error, *N*=3. Values with different letters in superscript differ at a significance level of 5%. T1-T4=raw whole milk treated for 2.5 (T1), 5 (T2), 7.5 (T3) and 10 (T4) min, T5-T8= skimmed milk treated for 2.5 (T5), 5 (T6), 7.5 (T7) and 10 (T8) min and T9-T12=skimmed bactofuged milk treated for 2.5 (T9), 5 (T10), 7.5 (T11) and 10 (T12) min. Experimental treatment: A (*P*=200 W, t=20 °C), B (P=200 W, t=55 °C), C (*P*=400 W, t=20 °C) and D (*P*=400 W, t=55 °C)

**Fig. 1:**
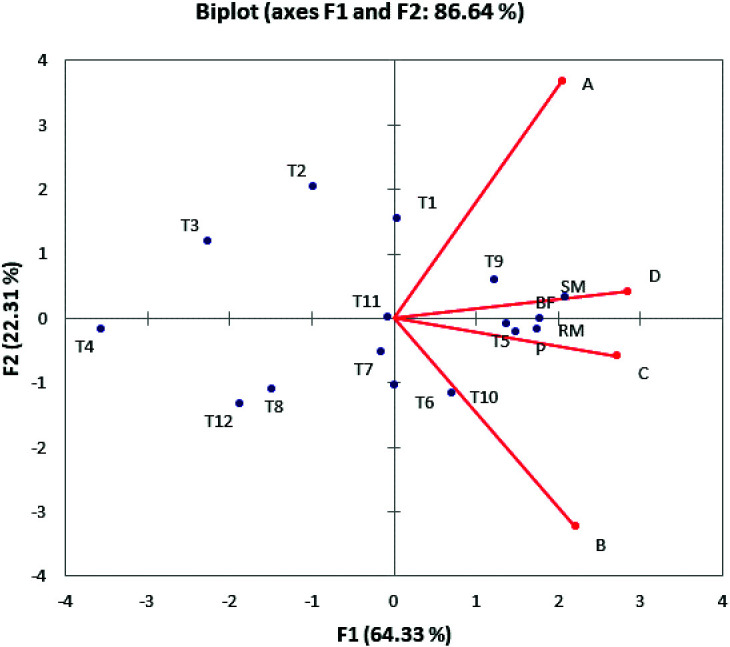
Principal component analysis for total sensory evaluation of samples (T1-T12). RM=raw whole milk, SM=skimmed milk, BF=skimmed bactofuged milk and P=pasteurized, T1-T4=raw whole milk treated for 2.5 (T1), 5 (T2), 7.5 (T3) and 10 (T4) min, T5-T8= skimmed milk treated for 2.5 (T5), 5 (T6), 7.5 (T7) and 10 (T8) min and T9-T12=skimmed bactofuged milk treated for 2.5 (T9), 5 (T10), 7.5 (T11) and 10 (T12) min, Experimental treatment: A (*P*=200 W, t=20 °C), B (*P*=200 W, t=55 °C), C (*P*=400 W, t=20 °C) and D (*P*=400 W, t=55 °C)

Namely, the total obtained scores of the sensory properties of the observed milk are lower than the reference untreated milk samples and their values decrease with time although there are no differences in the colour parameters and appearance among the samples. Total sensory evaluation of milk samples (Table 4) is the sum of all evaluated parameters (appearance, colour, odour and taste). In experiment A (200 W, 24 kHz, 20 °C), 81% of the samples received ≥15 points, and only 19% received <15 points, while in experiment C (400 W, 24 kHz, 20 °C), 56% samples received ≥15 and 44% received <15 points. The results show that the best process parameters to obtain acceptable organoleptic properties are power output of 200 W at 24 kHz and room temperature, while the lowest organoleptic properties were obtained using ultrasound power of 400 W at 24 kHz and room temperature. Since the frequency and temperature in both experiments were the same, the organoleptic characteristics were most affected by the power of ultrasound. The detailed analysis showed that treatment time is also one of the key parameters for good organoleptic parameters (*37*). The appearance and colour did not change significantly under the influence of ultrasound power, frequency, temperature and time. The odour changed considerably at higher ultrasound power (400 W), pasteurization at 72 °C and ultrasound treatment time of 7.5 and 10 min. Taste and total sensory evaluation of milk did not change significantly only under the following treatment conditions: at 20 and 55 °C and treatment time of 5 min and 15 s (pasteurization time). The taste was significantly affected by the power of 400 W at the frequency of 24 kHz. Taste and total sensory evaluation were greatly affected by a 7.5-minute treatment, and the treatment for 10 min had the strongest positive impact. This is in line with Juliano *et al.* (*38*), who showed that milk fat oxidation can be controlled by reducing the time and temperature of the probe. Jurić *et al.* (*37*) also determined the negative impact of the time of treatment on sensory properties. Of course, the effect of ultrasound on the oxidation of fat depends on the milk fat content. Skimmed milk produces fewer volatile degradation (vitamins) and oxidation (some flavours) products, thus these samples received better total sensory evaluation than whole milk samples. In order to carry out more detailed statistical analyses, the same treatment conditions in all experiments are presented as unified, where treatments T1-T4 indicate whole milk, treatments T5-T8 indicate skimmed milk, and T9-T12 treatments indicate bactofuged skimmed milk treated for 2.5, 5, 7.5 and 10 min, respectively. The main component analysis of the sensory characteristics of the milk (Fig. 1), after the Verimax rotation, showed the grouping of the bactofuged samples (BF, T9-T12) in the 1st and 2nd quadrants of the PCA system. This kind of arrangement indicates the uniformity of the sensory evaluation of the bactofuged samples, but the fact that they are not all in the 1st quadrant indicates that their mean sensory ratings are significantly different from the reference bactofuged milk that received 19 points. The bactofuged milk that was treated the longest (T12, 10 min) received the lowest average total sensory score (15.5 points).

Treatments B (Fig. 1) significantly contribute to the first major component and they clarify 64.33% variation (F1) in the observed data set, the remaining treatments A and D, explains 22.31% variations in the observed set of sensory milk ratings, which are also dominant in the second major component (F2). F1 and F2 together explain 86.64% variation in the observed set of sensory evaluation data depending on milk sample treatments, which is a very acceptable percentage and in accordance with previous study performed under realistic conditions (*24*). Regardless of the treatment, the grouping of the best-rated milk is important, namely non-treated (whole milk, skimmed milk and skimmed bactofuged milk) and pasteurized milk (P).

In order to test the relationship between the composition of milk samples from all experiments and their sensory properties, the results of Pearson's matrix correlation are presented separately for each experiment in Tables S.1-S.4. Although the results of experiment A suggest that there is no correlation among the sensory properties, milk composition and physical characteristics, they exist but are not significant (significance level 0.05). The protein mass fraction is in a positive correlation with titratable acidity, non-fat solids, lactose and Ca. Total solids are in a negative correlation with most parameters, with the exception of fat, which is understandable since the milk fat is part of the total solids.

**Fig. 2:**
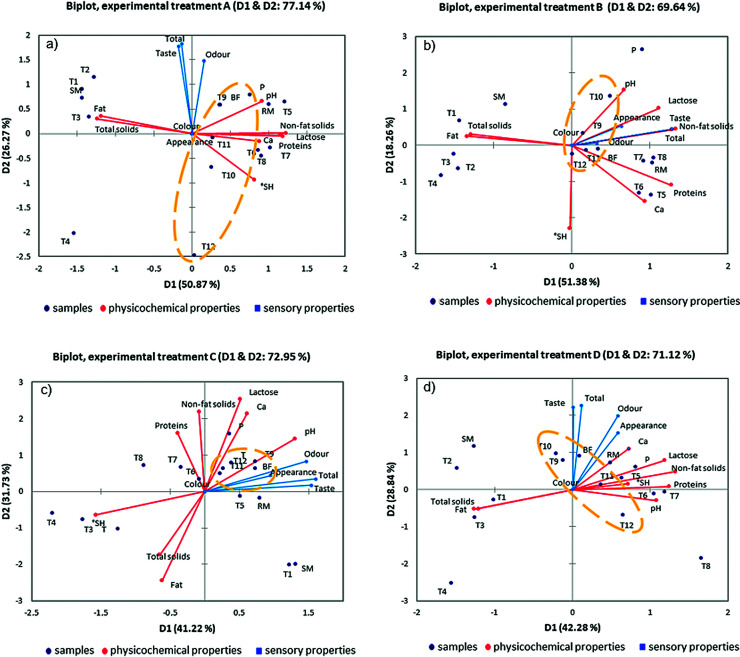
Biplot of the first two main components (D1 and D2) of the principal component analysis for physical and sensory properties of the treated milk samples: a) *P*_ultrasound_=200 W, *ν*=24 kHz, *t*=20 °C, b) *P*_ultrasound_=200 W, *ν*=24 kHz, *t*=55 °C, c) *P*_ultrasound_= 400 W, *ν*=24 kHz, *t*=20 °C, and d) *P*_ultrasound_=400 W, *ν*=24 kHz, *t*=55 °C. RM=raw whole milk, SM=skimmed milk, BF=skimmed bactofuged milk and P=pasteurized milk. A T1-T4=raw whole milk treated for 2.5 (T1), 5 (T2), 7.5 (T3) and 10 (T4) min, T5-T8= skimmed milk treated for 2.5 (T5), 5 (T6), 7.5 (T7) and 10 (T8) min and T9-T12=skimmed bactofuged milk treated for 2.5 (T9), 5 (T10), 7.5 (T11) and 10 (T12) min, Experimental treatment: A (*P*=200 W, *t*=20 °C), B (*P*=200 W, *t*=55 °C), C (*P*=400 W, *t*=20 °C) and D (*P*=400 W, *t*=55 °C)

However, the advantage of the principal component analysis (Fig. 2) is that it simultaneously shows the changes in sensory and physical characteristics of milk. Thus, it is possible to observe the potential grouping or separation of the treated milk samples against the bactofuged milk samples. Fig. 2 shows the principal component analysis for four different process conditions (A-D), where we observed the physical and sensory characteristics of the milk and the bactofuged samples grouped together in all experiments. Bactofuged samples are independent of the experimental data spread in different squares, but still make a group clearly separated from the other treatments. Under experiment conditions: 400 W, 24 kHz and 20 °C, the bactofuged samples form a very narrow group and are arranged exclusively in the first quadrant (Fig. 2c).

All variations in the observed data set ranged from 69.64% under experimental conditions of 200 W, 24 kHz and 55 °C (Fig. 2b) to 77.14% under the conditions of 200 W, 24 kHz and 20 °C (Fig. 2a). The higher percentage in all experiments belongs to the first major component (D1) dominated by the milk composition and physical characteristics observed in the different samples of milk. In Fig. 2b and Fig. 2c, the bactofuged samples are very close to the reference bactofuged sample. The proximity of treated milk sample and bactofuged milk sample indicates similarity in physicochemical composition and/or sensory evaluation. At 200 W, 24 kHz and 55 °C, total solids, non-fat solids, fat, proteins, lactose and Ca affected the taste (Fig. 2b). The average overall impression of bactofuged samples under these conditions received 12.43 points. Under experimental conditions of 400 W, 24 kHz and 20 °C, a high contribution of the titratable acidity, pH, odour, taste and total impression (Fig. 2c) was observed.

The worst total sensory score (Fig. 1 and Fig. 2) was found in bactofuged samples (score <15 points for 43.75% samples). Bactofuged milk samples received the worst overall sensory score (<15 points: 43.75% of the samples; Fig. 1 and Fig. 2). The reason for this can be seen from a comparative presentation of the results of the analysis of the main components (Fig. 2), where the samples of bactofuged milk were treated for a longer time than the reference untreated bactofuged sample.

## CONCLUSIONS

The results of the performed experiments and the multivariate statistical analysis revealed that the applied ultrasound power of 200 W, frequency of 24 kHz and the treatment time of the milk samples had no significant effect on the titratable acidity and pH of the milk. All the bactofuged samples of skimmed milk had lower values of total solids, non-fat solids, protein and calcium than the samples of the same batch and milk fat. Different conditions of high-power ultrasonic treatment did not affect the changes in total solids, non-fat solids, milk fat and protein. Temperature (20 and 55 °C) and shorter treatment time with ultrasound up to 5 min had no significant effect on the overall sensory evaluation of milk, while ultrasound power of 400 W had a significant effect on the change of taste of the whole milk samples treated for longer than 5 min. The best sensory characteristics of the bactofuged skimmed milk samples were obtained with an ultrasound power of 400 W for up to 7.5 min regardless of the temperature. For all milk types (whole milk, skimmed and skimmed bactofuged milk), the highest sensory evaluation was obtained with ultrasound power of 200 W, frequency of 24 kHz, temperature of 20 °C and treatment time of 7.5 min. Ultrasonic treatment of milk is a relatively inexpensive technology that rounds out flavour and aroma at much lower temperatures than pasteurization. Automated ultrasound systems that can be safely integrated into food production are still being developed.

## Figures and Tables

**Table S1:** Pearson correlation matrix, milk composition and sensory properties of milk samples in experimental treatment A (*P*=200 W, *ν*=24 kHz, *t*=20 °C)

Variable	Titratable acidity/°SH	pH	TS	SNF	Fat	Protein	Lactose	Ca	Appearance	Colour	Odour	Taste	Total sensory evaluation
°SH	**1**	0.1148	**-0.6892**	**0.5397**	**-0.6852**	**0.5549**	**0.5312**	0.3907	/	/	-0.2386	-0.4712	-0.4610
pH	0.1148	**1**	**-0.6869**	**0.5398**	**-0.6862**	0.4948	**0.5728**	0.3255	/	/	0.1782	0.2235	0.2289
TS	**-0.6892**	**-0.6869**	**1**	**-0.7651**	**0.9939**	**-0.7094**	**-0.8187**	-0.4641	/	/	0.0783	0.2830	0.2666
SNF	**0.5397**	**0.5398**	**-0.7651**	**1**	**-0.8310**	**0.9017**	**0.9340**	**0.6261**	/	/	0.1112	-0.1215	-0.0915
Fat	**-0.6852**	**-0.6862**	**0.9939**	**-0.8310**	**1**	**-0.7619**	**-0.8691**	**-0.4997**	/	/	0.0496	0.2627	0.2438
Protein	**0.5549**	0.4948	**-0.7094**	**0.9017**	**-0.7619**	**1**	**0.7109**	**0.8959**	/	/	0.1579	-0.1759	-0.1330
Lactose	**0.5312**	**0.5728**	**-0.8187**	**0.9340**	**-0.8691**	**0.7109**	**1**	0.3368	/	/	0.0259	-0.1128	-0.0972
Ca	0.3907	0.3255	-0.4641	**0.6261**	**-0.4997**	**0.8959**	0.3368	**1**	/	/	0.1397	-0.2357	-0.1896
Appearance	/	/	/	/	/	/	/	/	/	/	/	/	/
Colour	/	/	/	/	/	/	/	/	/	/	/	/	/
Odour	-0.2386	0.1782	0.0783	0.1112	0.0496	0.1579	0.0259	0.1397	/	/	**1**	**0.5917**	**0.6896**
Taste	-0.4712	0.2235	0.2830	-0.1215	0.2627	-0.1759	-0.1128	-0.2357	/	/	**0.5917**	**1**	**0.9918**
Total sensory evaluation	-0.4610	0.2289	0.2666	-0.0915	0.2438	-0.1330	-0.0972	-0.1896	/	/	**0.6896**	**0.9918**	**1**

Significance level of 5%. Bold values=significant correlation (p<0.05). TS=total solids, SNF=non-fat solids

**Table S2:** Pearson correlation matrix, milk composition and sensory properties of milk samples in experimental treatment B (*P*=200 W, *ν*=24 kHz, *t*=55 °C)

Variable	Titratable acidity/°SH	pH	TS	SNF	Fat	Protein	Lactose	Ca	Appearance	Colour	Odour	Taste	Total sensory evaluation
°SH	**1**	**-0.6003**	-0.0330	-0.1416	-0.0284	0.3655	-0.3386	**0.5229**	-0.0716	/	0.0444	-0.1734	-0.1643
pH	**-0.6003**	**1**	-0.2489	0.4827	-0.2697	0.2190	0.4886	0.1469	0.3742	/	0.2140	0.4031	0.4223
TS	-0.0330	-0.2489	**1**	**-0.7352**	**0.9943**	**-0.7866**	**-0.6319**	**-0.5648**	-0.3796	/	0.0324	**-0.6700**	**-0.6533**
SNF	-0.1416	0.4827	**-0.7352**	**1**	**-0.7979**	**0.6865**	**0.9414**	0.4532	0.3057	/	0.1874	**0.7687**	**0.7625**
Fat	-0.0284	-0.2697	**0.9943**	**-0.7979**	**1**	**-0.8023**	**-0.6947**	**-0.5709**	-0.3797	/	-0.0002	**-0.7099**	**-0.6940**
Protein	0.3655	0.2190	**-0.7866**	**0.6865**	**-0.8023**	**1**	0.4203	**0.9114**	0.2818	/	0.2124	**0.6739**	**0.6736**
Lactose	-0.3386	0.4886	**-0.6319**	**0.9414**	**-0.6947**	0.4203	**1**	0.1557	0.2662	/	0.0916	**0.6599**	**0.6485**
Ca	**0.5229**	0.1469	**-0.5648**	0.4532	**-0.5709**	**0.9114**	0.1557	**1**	0.0771	/	0.1510	0.3973	0.3945
Appearance	-0.0716	0.3742	-0.3796	0.3057	-0.3797	0.2818	0.2662	0.0771	**1**	/	-0.1240	0.3739	0.3991
Colour	/	/	/	/	/	/	/	/	/	/	/	/	/
Odour	0.0444	0.2140	0.0324	0.1874	-0.0002	0.2124	0.0916	0.1510	-0.1240	/	**1**	0.3051	0.3725
Taste	-0.1734	0.4031	**-0.6700**	**0.7687**	**-0.7099**	**0.6739**	**0.6599**	0.3973	0.3739	/	0.3051	**1**	**0.9962**
Total sensory evaluation	-0.1643	0.4223	**-0.6533**	**0.7625**	**-0.6940**	**0.6736**	**0.6485**	0.3945	0.3991	/	0.3725	**0.9962**	**1**

Significance level of 5%. Bold values=significant correlation (p<0.05). TS=total solids, SNF=non-fat solids

**Table S3:** Pearson correlation matrix, milk composition and sensory properties of l milk samples in experimental treatment C (*P*= 400 W, *ν*=24 kHz, *t*=20 °C)

Variable	Titratable acidity/°SH	pH	TS	SNF	Fat	Protein	Lactose	Ca	Appearance	Colour	Odour	Taste	Total sensory evaluation
°SH	**1**	**-0.8606**	0.4570	-0.0542	**0.5975**	0.0287	-0.4929	-0.4711	**-0.6283**	/	**-0.9245**	**-0.8168**	**-0.8860**
pH	**-0.8606**	**1**	**-0.5134**	0.2534	**-0.7918**	0.2039	**0.6937**	**0.6156**	**0.5652**	/	**0.8546**	**0.6935**	**0.7693**
TS	0.4570	**-0.5134**	**1**	**-0.8341**	**0.5200**	0.2628	**-0.5653**	**-0.6228**	-0.2267	/	-0.4599	-0.4222	-0.4487
SNF	-0.0542	0.2534	**-0.8341**	**1**	-0.4471	-0.0040	**0.6549**	**0.5335**	0.0288	/	0.1197	0.0633	0.0781
Fat	**0.5975**	**-0.7918**	**0.5200**	-0.4471	**1**	**-0.5889**	**-0.8523**	**-0.7578**	-0.3970	/	**-0.6137**	-0.3220	-0.4120
Protein	0.0287	0.2039	0.2628	-0.0040	**-0.5889**	**1**	**0.5494**	0.2545	0.0531	/	0.0192	-0.1975	-0.1499
Lactose	-0.4929	**0.6937**	**-0.5653**	**0.6549**	**-0.8523**	**0.5494**	**1**	**0.6561**	0.3278	/	**0.5239**	0.3810	0.4354
Ca	-0.4711	**0.6156**	**-0.6228**	**0.5335**	**-0.7578**	0.2545	**0.6561**	**1**	0.2085	/	**0.5287**	0.4219	0.4625
Appearance	**-0.6283**	**0.5652**	-0.2267	0.0288	-0.3970	0.0531	0.3278	0.2085	**1**	/	0.3952	0.3810	0.4444
Colour	/	/	/	/	/	/	/	/	/	/	/	/	/
Odour	**-0.9245**	**0.8546**	-0.4599	0.1197	**-0.6137**	0.0192	**0.5239**	**0.5287**	0.3952	/	**1**	**0.7889**	**0.8675**
Taste	**-0.8168**	**0.6935**	-0.4222	0.0633	-0.3220	-0.1975	0.3810	0.4219	0.3810	/	**0.7889**	**1**	**0.9885**
Total sensory evaluation	**-0.8860**	**0.7693**	-0.4487	0.0781	-0.4120	-0.1499	0.4354	0.4625	0.4444	/	**0.8675**	**0.9885**	**1**

5% significance level. Bold values: significant correlation (p<0.05). TS=Total solids, SNF=non-fat solids

**Table S4:** Pearson correlation matrix, milk composition and sensory properties of milk samples in experimental treatment D (*P*=400 W, *ν*=24 kHz, *t*=55 °C)

Variable	Titratable acidity/°SH	pH	TS	SNF	Fat	Protein	Lactose	Ca	Appearance	Colour	Odour	Taste	Total sensory evaluation
°SH	**1**	0.3383	-0.4058	0.2940	-0.4154	**0.5044**	0.1364	0.1326	0.3466	/	0.3720	0.0472	0.1101
pH	0.3383	**1**	-0.4310	**0.6122**	-0.4696	**0.5874**	**0.5082**	0.3760	0.3461	/	0.1993	-0.0978	-0.0386
TS	-0.4058	-0.4310	**1**	**-0.7340**	**0.9960**	**-0.6689**	**-0.7384**	-0.3291	-0.3585	/	**-0.5412**	-0.2414	-0.3016
SNF	0.2940	**0.6122**	**-0.7340**	**1**	**-0.7900**	**0.8365**	**0.9221**	**0.6448**	0.4238	/	0.4826	0.2251	0.2833
Fat	-0.4154	-0.4696	**0.9960**	**-0.7900**	**1**	**-0.7203**	**-0.7776**	-0.3731	-0.3817	/	**-0.5513**	-0.2483	-0.3102
Protein	**0.5044**	**0.5874**	**-0.6689**	**0.8365**	**-0.7203**	**1**	**0.5842**	0.2526	0.3743	/	0.3342	0.1226	0.1718
Lactose	0.1364	**0.5082**	**-0.7384**	**0.9221**	**-0.7776**	**0.5842**	**1**	**0.7671**	0.4538	/	**0.5647**	0.2890	0.3512
Ca	0.1326	0.3760	-0.3291	**0.6448**	-0.3731	0.2526	**0.7671**	**1**	**0.5603**	/	**0.5421**	0.3285	0.3883
Appearance	0.3466	0.3461	-0.3585	0.4238	-0.3817	0.3743	0.4538	**0.5603**	**1**	/	**0.7474**	0.4809	**0.5723**
Colour	/	/	/	/	/	/	/	/	/	/	/	/	/
Odour	0.3720	0.1993	**-0.5412**	0.4826	**-0.5513**	0.3342	**0.5647**	**0.5421**	**0.7474**	/	**1**	**0.7417**	**0.8178**
Taste	0.0472	-0.0978	-0.2414	0.2251	-0.2483	0.1226	0.2890	0.3285	0.4809	/	**0.7417**	**1**	**0.9918**
Total sensory evaluation	0.1101	-0.0386	-0.3016	0.2833	-0.3102	0.1718	0.3512	0.3883	**0.5723**	/	**0.8178**	**0.9918**	**1**

5% significance level, Bold values: significant correlation (p<0.05). TS=Total solids, SNF=non-fat solids
